# Case Report: A Novel Splice-Site Mutation in DNAJB6 Associated With Juvenile-Onset Proximal–Distal Myopathy in a Chinese Patient

**DOI:** 10.3389/fgene.2022.925926

**Published:** 2022-06-23

**Authors:** Guang Ji, Ning Wang, Xu Han, Yaye Wang, Jinru Zhang, Yue Wu, Hongran Wu, Shaojuan Ma, Xueqin Song

**Affiliations:** ^1^ Department of Neurology, Second Hospital of Hebei Medical University, Shijiazhuang, China; ^2^ Neurological Laboratory of Hebei Province, Shijiazhuang, China

**Keywords:** DNAJB6, LGMD1D, G/F domain, splice-site mutation, de novo, rimmed vacuoles

## Abstract

DNAJB6 was identified as the causative gene of limb-girdle muscular dystrophy type 1D. In recent years, the phenotypic and molecular spectrum of DNAJB6-myopathy has been expanded, and several mutations of DNAJB6 have been identified in Europe, North America, and Asia. Interestingly, almost all identified mutations in previous reports were point mutations, and most of them were clustered in exon 5, which encodes the G/F domain of DNAJB6. The so-far unique splice site mutation eliminating the entire G/F domain was reported to cause a severe, early-onset phenotype. Here, we report a juvenile-onset Chinese patient who presented with proximal–distal myopathy as well as esotropia and facial weakness. Muscle pathology showed rimmed vacuolation and myofibrillar disarrangement. A novel splice-site mutation NM_058246:c.236-1_240delGGTGGA of the DNAJB6 gene was identified by targeted exome sequencing, which results in a severe defect of the G/F domain. This rare mutation type expands the molecular spectrum of DNAJB6-myopathy and further underlines the importance of the G/F region.

## Introduction

The DNAJB6 gene encodes the DNAJB6 protein, which is a member of the heat shock protein (HSP) 40 family of co-chaperones, interacting with the chaperone of HSP70 through their J domains (Kampinga and Craig, 2010). There are two isoforms of DNAJB6 distinguished by their C-terminal parts: DNAJB6a and DNAJB6b (Sarparanta et al., 2020). Both isoforms of DNAJB6 harbor the J domain at the N-terminus, the glycine/phenylalanine-rich (G/F) region, and a serine/threonine-rich (S/T) region that recognizes and binds to the substrates. DNAJB6 is ubiquitously expressed and is of great importance in neurodegenerative diseases by inhibiting the aggregation of misfolded proteins, such as α-synuclein, TDP-43, polyglutamine-containing huntingtin, Aβ42, etc. (Chuang et al., 2002; Sarparanta et al., 2012; Mansson et al., 2014; Stein et al., 2014). DNAJB6 was identified as the pathogenic gene of limb-girdle muscular dystrophy type 1D (LGMD1D) in 2012 (Harms et al., 2012; Sarparanta et al., 2012), which was characterized by an autosomal dominant–inherited, progressive proximal muscular weakness. Pathologically, all reported muscular disorders associated with DNAJB6 mutations have exhibited common histological features characterized by sarcomeric protein aggregation, autophagic vacuolization, and myofibrillar degeneration, so the DNAJB6 myopathy was also classified as myofibrillar myopathy for pathological classification.

When identified, DNAJB6 myopathy was originally known as a late-onset, slowly progressive disease, and most patients can keep walking independently even at old age. However, with the discovery of novel mutations, some mutations were found to cause an earlier, more severe, or distal-onset disease (Harms et al., 2012; Nam et al., 2015; Palmio et al., 2015; Ruggieri et al., 2015; Monies et al., 2016; Tsai et al., 2017; Kim et al., 2018). In recent years, several DNAJB6 mutations were identified in different regions, and the patients were mainly reported in Europe (Palmio et al., 2015; Ruggieri et al., 2015; Jonson et al., 2018) and North America (Suarez-Cedeno et al., 2014; Ruggieri et al., 2015; Nallamilli et al., 2018). In Asia, mutations causing DNAJB6 myopathy have been reported in Saudi Arabia (Monies et al., 2016; Bohlega et al., 2018), Korea (Nam et al., 2015; Kim et al., 2018), and Japan (Sato et al., 2013). For people of Han Chinese origin, a heterozygous mutation (p.Pro96Leu) in the DNAJB6 gene was first identified in a Taiwanese family (Tsai et al., 2017). Interestingly, regardless of regions and ethnic groups, the mutations reported before were clustered in exon 5, which encodes the G/F domain of DNAJB6, except for only two mutational sites in the DNAJB6 J domain identified as the causative gene of the disease (Palmio et al., 2020). Furthermore, almost all identified mutations in previous reports were point mutations, and the so-far unique splice site mutation eliminating the entire G/F domain which caused a severe, early-onset phenotype was reported in 2015 (Ruggieri et al., 2015). Here, we report a juvenile-onset Chinese patient who presented with a proximal–distal phenotype associated with a novel alternative splice-site mutation which leads to complete deletion or partial deletion of exon 5. This rare mutational type expands the molecular spectrum of DNAJB6-myopathy which further underlines the importance of the G/F region.

## Case Description

### Clinical Data

A 17-year-old female complained of fatigue for 2 years, especially in the lower limbs. She experienced weakness when squatting and climbing stairs, without disturbance when walking or performing daily activities involving the upper limbs. In the recent 3 months, the symptoms progressed, and she felt easy to trip and needed help in climbing upstairs. The patient had visited a primary hospital and performed an MRI of the brain and knee joints examination. The brain MRI was normal, but the knee joints MRI showed a small amount of effusion in the right knee cavity and atrophy of the right thigh and calf muscles; however, a definite diagnosis was not made, and the patient did not receive any therapeutic intervention (Table 1). Her parents and a 14-year-old brother showed no similar symptoms. The patient achieved normal developmental milestones and had no special past medical history but esotropia since childhood.

**TABLE 1:** The timeline with relevant data from the episode of care.

Time	Episode of Care	Examination and Treatment
November 2018	Fatigue in lower limbs and weakness when squatting and climbing stairs	None
August 2020	Symptoms progressed, easy to trip, and need help in climbing upstairs	MRI of the brain and the knee joints was performed, without appropriate treatment
November 2020	Visit our hospital	Muscle biopsy and gene sequencing

**FIGURE 1:**
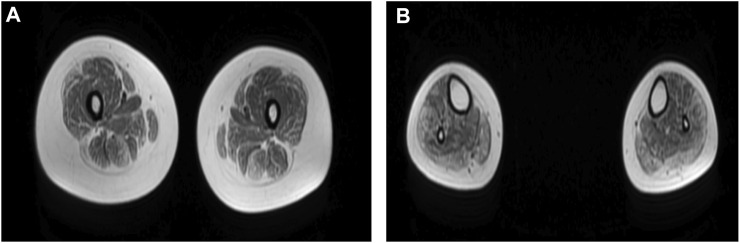
**(A,B)** Muscle MRI of lower limbs showed fatty infiltration and muscle atrophy of proximal and distal lower limb muscles, with the most prominent involvement in posterior muscles. **(A)** Thigh level. The biceps femoris (long head), semitendinosus, vastus lateralis, gracilis, and sartorius muscle are mildly to moderately affected. The rectus femoris is the best spared muscle. **(B)** Calf level. The gastrocnemius (medial head and lateral head), peroneus (fibularis) brevis, and peroneus (fibularis) longus muscle are moderately affected.

Neurological examination showed weakness of the upper and lower limbs, with both proximal and distal involved and the left side slightly more severe than the right. Atrophy of bilateral thenar and hypothenar could be observed. It also revealed bilateral esotropia and facial weakness. Ptosis, diplopia, and bulbar features were absent. Laboratory tests showed normal levels of serum CK. Electromyography showed myogenic damage with normal nerve conduction velocity. Electrocardiogram, cardiac ultrasonography, and lung function were normal. MRI of the muscles in the lower limbs showed fatty infiltration and muscle atrophy of the lower limb muscles, most prominent involvement in posterior muscles (Figure 1).

### Pathological Findings

After informed consent, a muscle biopsy was performed on the left biceps brachii muscle of the patient. Histochemical staining of hematoxylin-eosin (HE) and modified Gomori trichrome (MGT) were processed according to traditional methods on frozen muscle sections. For immunohistochemical analysis, the following primary antibodies were applied to the muscle sections: desmin (ab8976, Abcam), p62 (P0067, Sigma-Aldrich), TDP-43 (12892-1-AP, Proteintech), LC3B (L7543, Sigma-Aldrich), and LAMP2 (#49067S, Cell Signaling Technology). Images were acquired using a light microscope (Olympus BX51, United States). For electron microscopy, 80 nm ultrathin sections were made, and images were acquired on a transmission electron microscope (TEM, JEM 1230, JEOL).

**FIGURE 2:**
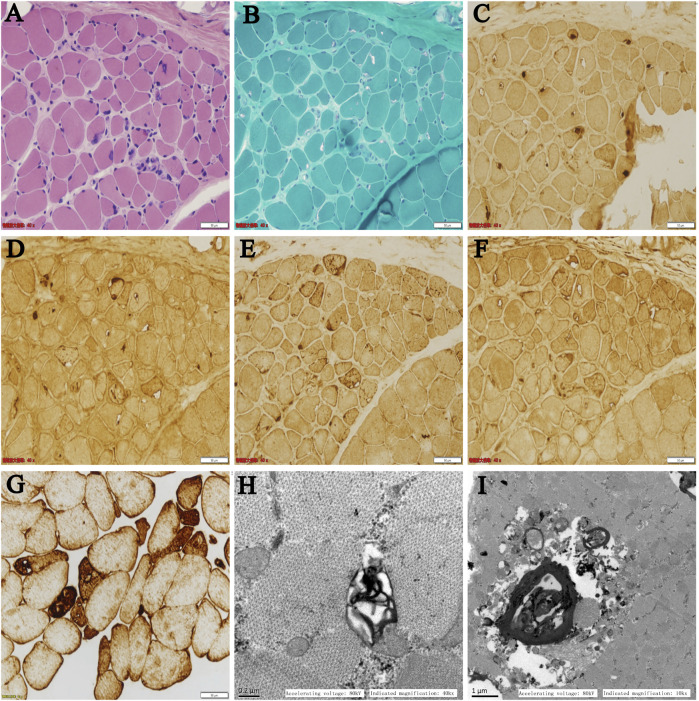
**(A–F)** Serial sections from the patient’s left biceps brachii muscle on light microscopy: **(A)** HE and **(B)** MGT staining revealed mildly proliferated connective tissue and fiber size variation with atrophic fibers. Rimmed vacuoles were found in a portion of the fibers. Immunohistochemistry showed positive reactivity for **(C)** TDP-43, **(D)** p62, and **(E)** LAMP2 in the rimmed vacuoles. **(F)** LC3 reactivity was absent or less abundant in the rimmed vacuolar regions. **(G)** Desmin reactivity showed moderate or strong expression in rimmed vacuolated fibers. **(H,I)** Ultrastructural changes on electron microscopy: **(H)** myelin bodies and **(I)** destroyed myofibrillar structure replaced by loop debris materials were found on transverse sections.

On light microscopy, HE and MGT staining (Figure 2A,B) revealed mildly proliferated connective tissue and adipose tissue in the perimysium and endomysium and fiber size variation with atrophic fibers. Rimmed vacuoles were found in a small portion of fibers. The eosinophilic cytoplasmic material deposition was also found in some fibers. On immunohistochemistry, the rimmed vacuoles showed positive reactivity for TDP-43, p62, and LAMP2 (Figure 2C–E), whereas components of LC3 reactivity were absent or less abundant in the rimmed vacuolar regions (Figure 2F), indicating impaired autophagic flux and defect degradation. Desmin reactivity showed moderate or strong expression in rimmed vacuolated fibers (Figure 2G). On electron microscopy, myelin bodies were found (Figure 2H), and myofibrillar structure was destroyed and replaced by loop debris materials in some regions (Figure 2I).

### Targeted Exome Sequencing, Transcript Sequencing, and Molecular Modeling

**FIGURE 3:**
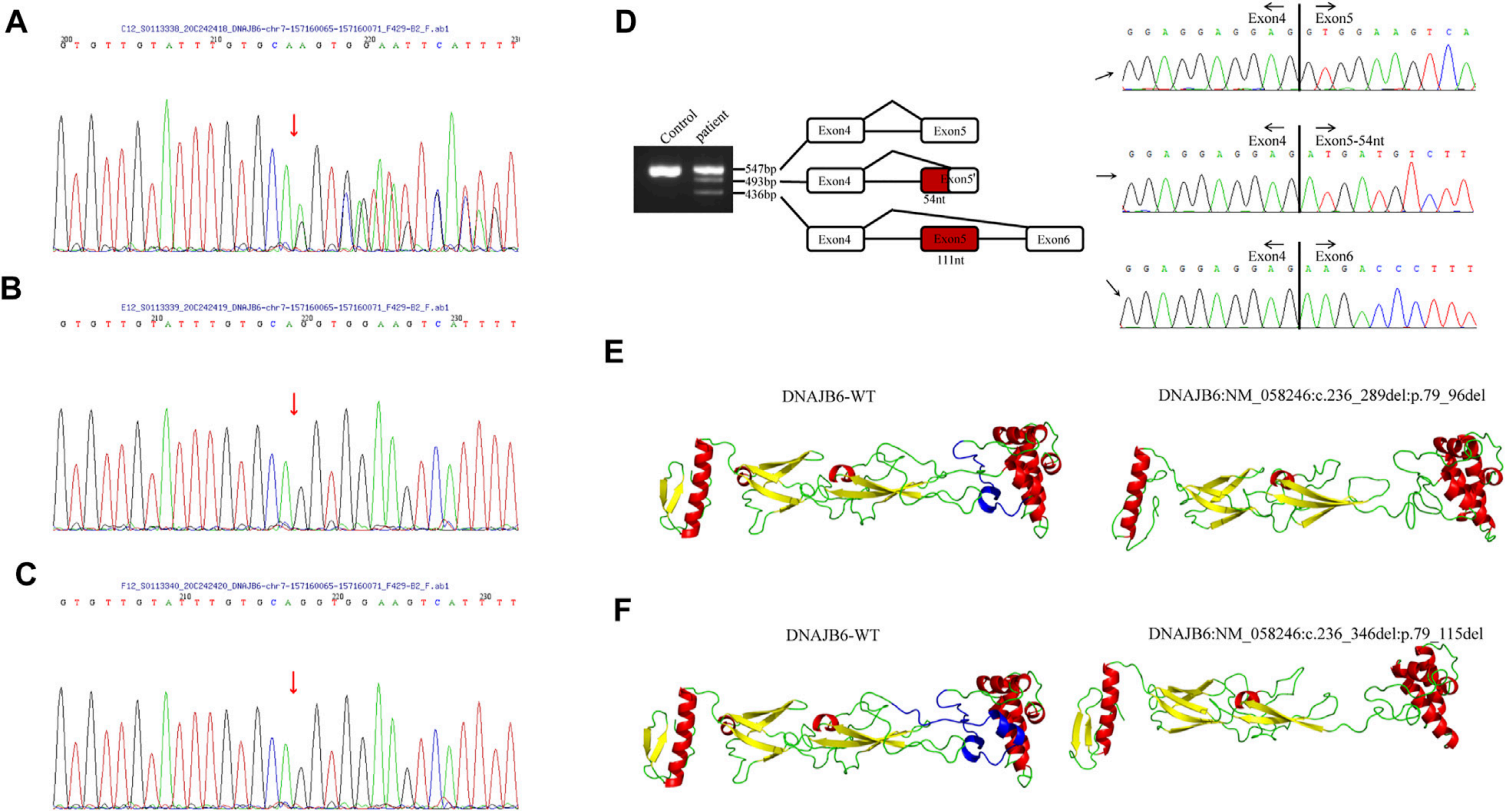
**(A–C)** The results of Sanger sequencing of the patient and her parents. **(A)** A heterozygous mutation c.236-1_240delGGTGGA (p.G79Efs*4) of the DNAJB6 gene was identified, and the mutational site of the patient was confirmed by Sanger sequencing. Sanger verification of her father **(B)** and mother **(C)** showed that this site was normal. **(D)**. DNAJB6 transcript sequencing: Gel electrophoresis of the patient showed three different size bands, namely, 547, 493, and 436 bp. The 547 bp band is of the same size as the control one and is a wild-type transcript. By sequencing, the 493 bp band refers to 54-nucleotide deletion at the beginning of exon 5, and the 436 bp band refers to the whole 111-nucleotide delete of exon5, which encodes the G/F domain. **(E,F)** I-TASSER software was used to develop a suitable model to simulate the effect of the mutation region; the 3D protein modeling predicted that p.79_96del mutation would result in the loss of an α-helix **(E)** and p.79_115del mutation would result in the loss of two α-helices **(F)**.

Informed consent was signed by the patient’s parent for genetic analysis, and the peripheral blood of the patient and her parents was collected. Clinical exome sequencing analysis was accomplished by MyGenostics Inc., Beijing, China. A heterozygous mutation NM_058246:c.236-1_240delGGTGGA of the DNAJB6 gene was identified within exon 5 of DNAJB6 by genetic testing. The mutational site was confirmed by Sanger sequencing, and this site was normal in her parents (Figure 3A–C).

To verify the effect of variation on mRNA splicing, total RNA from the patient’s muscle tissues was extracted, and reverse transcription was performed. The RT-PCR products were separated by electrophoresis analysis and identified by TA cloning. Gel electrophoresis of the patient showed three different size bands, namely, 547, 493, and 436 bp (Figure 3D). The 547 bp band is of the same size as the control one and is a wild-type transcript. By sequencing, the 493 bp band refers to 54-nucleotide deletion at the beginning of exon 5, and the 436 bp band refers to the whole 111-nucleotide deletion of exon 5, which encodes the G/F domain (Figure 3D).

The I-TASSER software was used to develop a suitable model to simulate the effect of the mutation region (Zhang, 2008; Yang et al., 2015). The 3D protein modeling predicted that p.79_96del mutation would result in the loss of an α-helix (Figure 3E) and p.79_115del mutation would result in the loss of two α-helices (Figure 3F).

## Discussion

### Clinical Manifestation

DNAJB6 deficiency has been found to be associated with LGMD1D, a muscular disorder clinically characterized by slowly progressive proximal, limb-girdle muscle weakness. In recent years, however, mutations in the DNAJB6 gene have been reported to cause a wide spectrum of phenotypes based on the age of the onset, the severity of involvement, and the affected group of muscles. While most of the mutations have been reported to lead to a limb-girdle phenotype, some mutations were found to cause a distal myopathy or proximo-distal phenotype (Ruggieri et al., 2016; Sarparanta et al., 2020). The patient in this case also manifested a proximal–distal phenotype. Besides limb weakness, involvement of respiratory and bulbar muscles has been reported in some severe cases (Ruggieri et al., 2016), and facial weakness was also common in DNAJB6 myopathy (Ruggieri et al., 2015). Extraocular muscles have been spared in reported cases until now. Interestingly, esotropia was found in our patient, but the possibility that it was rather caused by extraocular muscles involvement in DNAJB6 myopathy or just a complicated disease remains unclear. Further MRI of the extraocular muscles of this patient was not performed, which may help distinguish the two situations.

### Pathological Changes

On the pathological level, mutations in the DNAJB6 gene caused similar histological changes, including myofibrillar degeneration, protein accumulation, and autophagic vacuolation. Early changes in the pathology of DNAJB6 myopathy contain central myofibrillar lesions and Z-disc streaming followed by myofibrillar disintegration, and autophagic vacuoles appeared at later stages (Sandell et al., 2016). On light microscopy, DNAJB6 myopathy showed dystrophic or myopathic changes accompanied by rimmed vacuolation and myofibrillar aggregation in muscle pathology. This case showed definite rimmed vacuolar pathology; besides, eosinophilic cytoplasmic material deposition and the positive reactivity of desmin were also observed, which suggest myofibrillar aggregation. A previous study showed that the rimmed vacuoles were reactive for markers of impaired autophagy and defect degradation, such as LC3, p62, TDP-43, ubiquitin, and SMI-31 but do not stain for LAMP2, a marker of autophagosome–lysosome fusion (Sandell et al., 2016). However, the rimmed vacuoles in our patient were reactive for TDP-43, p62, and LAMP2, whereas the autophagosome marker LC3 reactivity was absent or less abundant, which was different from the previous study, suggesting that a variation in autophagy markers may appear in different patients or at different stages of the disease. On ultrastructure, these myofibrillar inclusions contained an abnormal accumulation of several proteins, such as myotilin, αB-crystallin, and desmin, and showed extensive myofibrillar disorganization with a mass of the Z-disk material. Pronounced larger pleomorphic myofibrillar aggregates were found in the later process of rare cases as major changes in rimmed vacuolar pathology (Sandell et al., 2016). Myelin bodies and myofibrillar disorganization were found in this case; however, large loop debris materials have not been defined, which may be formed by myofibrillar aggregation.

### The Spectrum of Mutations

Until recently, 18 pathogenic mutations have been identified in DNAJB6 (Sarparanta et al., 2020). Interestingly, almost all identified mutations were clustered within exon 5 of the gene encoding a short stretch of amino acids in the G/F region, and only two pathogenic mutations (p.Ala50Val and p.Glu54Ala) in the J domain of DNAJB6 have been reported to cause distal or proximo-distal myopathies until now (Palmio et al., 2020), highlighting the G/F region as a mutational hot spot. Besides the patient in our report, another splice site mutation (c.346+5G>A) causing exon 5 skipping that eliminates the entire G/F domain has been reported, which further underlined the importance of the G/F region (Ruggieri et al., 2015). Currently, it is believed that the DNAJB6 G/F spiral folds play a critical role in the contact with the J domain (Ruggieri et al., 2015). By protein modeling, we predicted that the mutation in our patient would result in the loss of one or two α-helices of DNAJB6, showing evidence that the mutation in this patient may also act through disturbing these contacts, which is consistent with the previously reported DNAJB6 mutations (Ruggieri et al., 2015). However, the direct effect of the G/F domain defect on DNAJB6 myopathy is not clear. In addition, compared with the patient with a mutation that eliminates the entire G/F domain reported before, our patient developed symptoms later and presented with a proximal–distal phenotype without severe symptoms such as respiratory and bulbar involvement. This may be due to the alternative splicing only causing a small amount of abnormal mRNA, and the DNAJB6 protein, at least a part of it, still functions.

In addition, it has been known that LGMD1D was autosomal-dominantly inherited myopathy caused by gain-of-deleterious-function mutations in the DNAJB6 gene, and DNAJB6b was known as the pathogenic isoform. Interestingly, however, a homozygous mutation (p.Val232Gly fs*7) that localized to exon 9 of the DNAJB6 gene was reported to cause an autosomal-recessively inherited late-onset very recently (Qian et al., 2021). The novel mutation exclusively caused loss of the “a” region in DNAJB6a and resulted in a recessive toxic effect. These new findings uncovered the pathogenic role of DNAJB6a deficiency and indicated that many unclear issues remained to be studied on DNAJB6.

In summary, DNAJB6 myopathy is a rare muscular disorder with varying clinical phenotypes such as the age of the onset, the severity of disease, and the muscles involved. Although this disease is mainly characterized by slowly progressive proximal, limb-girdle muscle weakness, a distal myopathy or proximo-distal phenotype should also be noticed, especially when the rimmed vacuoles and myofibrillar aggregation were found in muscle pathology. Gene sequencing may help differentiate this from other muscular diseases.

## Data Availability

The datasets for this article are not publicly available due to concerns regarding participant/patient anonymity. Requests to access the datasets should be directed to the corresponding author.
